# Within‐Person Changes in Daily Ovarian Hormone Levels Influence Genetic Effects on Emotional Eating in Women

**DOI:** 10.1002/eat.24545

**Published:** 2025-09-17

**Authors:** Megan E. Mikhail, Kristen M. Culbert, Pamela K. Keel, S. Alexandra Burt, Cheryl L. Sisk, Alexander Johnson, Steven Boker, Micheal C. Neale, Kelly L. Klump

**Affiliations:** ^1^ Department of Psychology Michigan State University East Lansing Michigan USA; ^2^ Department of Psychiatry and Behavioral Sciences University of California San Francisco California USA; ^3^ Department of Psychology Florida State University Tallahassee Florida USA; ^4^ Neuroscience Program Michigan State University East Lansing Michigan USA; ^5^ Department of Psychology University of Virginia Charlottesville Virginia USA; ^6^ Departments of Psychiatry, Human Genetics, and Psychology Virginia Commonwealth University Richmond Virginia USA

**Keywords:** binge eating, emotional eating, estradiol, ovarian hormones, progesterone, twin models, within‐person

## Abstract

**Introduction:**

Ovarian hormones (i.e., estradiol, progesterone) show robust phenotypic associations with binge eating and related behaviors (e.g., emotional eating) in females. Recent research suggests these associations may be due to ovarian hormone effects on genetic and environmental influences on dysregulated eating. However, no research has yet examined how within‐person fluctuations in hormone levels impact genetic/environmental influences on day‐to‐day changes in dysregulated eating from an individual's own mean. This omission is critical because phenotypic associations between ovarian hormones and dysregulated eating are strongest at a within‐person level in adult women, suggesting potentially unique and important within‐person effects.

**Method:**

Participants from same‐sex female twin pairs (*N* = 468) from the MSU Twin Registry provided measures of ovarian hormones and emotional eating daily for 49 consecutive days. We used continuous twin moderation models to examine how within‐person centered estradiol and progesterone impacted genetic/environmental influences on within‐person shifts in emotional eating.

**Results:**

Within‐person fluctuations in estradiol and progesterone significantly moderated genetic influences on within‐person changes in emotional eating. Stronger genetic influences were observed when the ratio of within‐person estradiol to progesterone was in the moderate range.

**Conclusion:**

Cyclic, within‐person changes in ovarian hormones may contribute to phenotypic changes in dysregulated eating across the menstrual cycle by dynamically regulating expression of underlying genetic risk. Genetic influences may be particularly pronounced under the hormonal conditions characteristic of the mid‐luteal phase (i.e., moderate estradiol and progesterone).

**Summary**

- Within‐person fluctuations in ovarian hormones are associated with daily phenotypic changes in dysregulated eating across the menstrual cycle.
- These changes may be driven by hormonal activation of genetic influences; however, until recently, models were unable to examine within‐person moderation of genetic effects.
- We found that genetic influences on within‐person changes in emotional eating were greatest when the ratio of estradiol to progesterone was moderate.
- Increased dysregulated eating during the mid‐luteal phase may be driven by hormonally mediated activation of genetic influences.

## 1 Introduction

Ovarian hormones show robust phenotypic associations with dysregulated eating in females (e.g., binge eating, or eating a large amount in a short time with loss of control; emotional eating, or eating in response to negative feelings). Estradiol is generally protective against these behaviors. Female rats that have undergone ovariectomy, which removes the body's primary source of estradiol, experience increased binge‐like eating (Klump et al. 2011), while administration of exogenous estradiol reverses this effect (Micioni Di Bonaventura et al. 2017). In women, dysregulated eating is lower during menstrual cycle phases when estradiol is high and progesterone is relatively low (i.e., late follicular phase, ovulation) (Edler et al. 2007; Klump et al. 2008, 2013, 2014). Conversely, progesterone appears to increase the risk for dysregulated eating (Edler et al. 2007; Klump et al. 2008) by antagonizing estradiol's protective effects (Klump et al. 2013, 2014).

Ovarian hormones may impact dysregulated eating by regulating the expression of genetic risk. The genes that influence ovarian hormone levels themselves are not directly associated with dysregulated eating (Klump et al. 2015, 2016). Instead, ovarian hormones likely affect eating behavior through the downstream transcriptional actions of estradiol and progesterone receptors on other neurobiological systems (e.g., serotonergic and dopaminergic systems; Östlund et al. 2003). While the specific genes that contribute to dysregulated eating are not yet fully characterized, evidence implicates genes involved in appetite regulation (e.g., FTO as identified in a recent GWAS; Termorshuizen et al. 2025), mood, and reward processing, all of which are regulated by estrogen and progesterone receptors (Hernández‐Hernández et al. 2019; Zhang et al. 2012). By modulating the expression of these genes, ovarian hormones may enhance or attenuate the behavioral manifestation of genetic predispositions, contributing to both stable individual differences and within‐person fluctuations in dysregulated eating.

While it is difficult to directly measure changes in gene expression in the brain in humans, twin designs offer an indirect method to quantify differences in genetic (and environmental) influences across ovarian hormone levels. Inferences regarding genetic influences in twin designs draw on the fact that monozygotic (MZ) twins share 100% of their DNA, while dizygotic (DZ) twins share 50% of their DNA on average. If MZ twins are more similar than DZ twins, this implies the presence of genetic influences. Extending the twin design a step further, if the difference in similarity between MZ and DZ twins varies across a moderator (e.g., ovarian hormone levels), this implies differential genetic influences at different levels of the moderator.

Past twin work from our lab supports the presence of genetic moderation effects for dysregulated eating. The heritability of emotional eating is significantly greater in the high‐risk mid‐luteal phase of the menstrual cycle as compared to lower risk phases (e.g., pre‐ovulation) (Klump et al. 2015) and in twins with higher versus lower mean progesterone levels (Klump et al. 2016). Interestingly, we found no changes in additive genetic effects across estradiol levels, but significant increases in shared environmental influences (i.e., environmental factors that increase co‐twin similarity) that may underlie estrogen's protective effects via novel non‐genomic (e.g., membrane estrogen receptors) or even genomic (e.g., regulation of highly conserved genes involved in nutrition, metabolism) pathways (see Klump et al. 2024).

However, an important limitation of prior work is the level of analysis; all models used mean and/or raw, absolute daily ovarian hormone levels (Klump et al. 2015, 2016, 2024). In adult women, stronger phenotypic associations between ovarian hormones and dysregulated eating have been observed at a within‐person level, where changes in ovarian hormones from an individual's own mean predict changes in dysregulated eating. Until recently, twin models were unable to model daily changes in etiologic influences across repeated measurements, making it difficult to examine within‐person effects of hormones on genetic/environmental influences over time. Twin models using raw, absolute levels of ovarian hormones and dysregulated eating cannot distinguish effects of between‐person individual differences from those of within‐person fluctuations across the menstrual cycle. Targeted within‐person models are needed to disentangle these effects and understand how changes in hormones from a person's own baseline may impact changes in their eating behavior. Understanding daily hormone‐driven shifts in risk could help women identify predictable windows of heightened vulnerability, clarify the factors underlying this vulnerability, and ultimately inform the development of targeted and personalized interventions.

The aim of the current study was therefore to investigate, for the first time, within‐person ovarian hormone effects on the genetic/environmental influences shaping day‐to‐day fluctuations in dysregulated eating. We hypothesized that genetic influences on emotional eating would be greatest during hormonal milieus replicating the high‐risk mid‐luteal phase (i.e., high progesterone, moderate estradiol). Analyses were conducted using our archival sample of 468 postmenarcheal female twins who provided hormone samples and emotional eating ratings daily for 45 consecutive days. This is the same sample leveraged in our previous work using mean and raw, absolute daily hormone values (Klump et al. 2016, 2024). To our knowledge, this is the only twin sample that has the requisite data (i.e., numerous daily measurements) to model within‐person effects.

## 2 Method

### 2.1 Participants

Analyses included 468 female twins ages 15–25 (M_age_ = 17.83, SD = 1.79) from same‐sex pairs recruited through the Michigan State University Twin Registry (MSUTR; Burt and Klump 2019) from 2007 to 2013 for the Twin Study of Hormones and Behavior Across the Menstrual Cycle (HBMC; Klump et al. 2013). Twins are identified for recruitment into the MSUTR through birth records and response rates are similar to other twin registries (Burt and Klump 2019). Because HBMC focused on ovarian hormone effects on dysregulated eating across the menstrual cycle, inclusion criteria included: (1) menstruation every 22–32 days for the past 6 months (indicating established, regular cycles and increasing the likelihood of at least one ovulatory cycle during the study); (2) no hormonal contraceptive use in the past 3 months; (3) no psychotropic or steroid medications in the past 4 weeks; (4) no pregnancy or lactation in the past 6 months; and (5) no history of genetic/medical conditions known to influence hormone functioning or appetite/weight (e.g., polycystic ovary syndrome) (Klump et al. 2013). HBMC participants did not differ from other MSUTR twins on disordered eating symptoms (e.g., emotional eating, body dissatisfaction; *d*s = 0.02–0.18) (Klump et al. 2013) and were demographically representative of Michigan with respect to race and ethnicity (See Table 1).

**TABLE 1:** Participant descriptive statistics (*N =* 468 twins).

Participant variable	Mean (SD) or % of Sample (*N*)
Age	17.83 (1.79) [range = 15 to 25]
Sex on birth certificate	
Female	100% (468)
Zygosity	
Twins from complete monozygotic (MZ) pairs	53.8% (252)
Twins from complete dizygotic (DZ) pairs	42.7% (200)
Twins without cotwin data	3.4% (16)
Racial identity	
White	78.6% (368)
Black/African American	10.9% (51)
Asian/Asian American	0.4% (2)
American Indian/Alaska Native	0.4% (2)
More than one race	4.7% (22)
Other/unknown	4.9% (23)
Ethnicity	
Hispanic/Latinx	9.0% (42)
Parental income	
Under $20,000	5.1% (24)
$20,000–$40,000	13.7% (64)
$40,000–$60,000	23.3% (109)
$60,000–$100,000	32.7% (153)
Over $100,000	20.9% (98)
Unknown/not reported	4.3% (20)
Daily estradiol rolling average (pg/mL)	2.90 (1.68) [range = 0.21 to 47.62]
Mean estradiol (pg/mL)	2.87 (1.43) [range = 0.46 to 12.48]
Within‐person centered estradiol (pg/mL)	0.00 (0.93) [range = −10.00 to 35.14]
Daily progesterone rolling average (pg/mL)	121.33 (86.79) [range = 6.80 to 648.63]
Mean progesterone (pg/mL)	120.59 (66.96) [range = 18.36 to 396.23]
Within‐person centered progesterone (pg/mL)	0.00 (55.20) [range = −202.26 to 412.75]
Daily emotional eating	0.31 (0.46) [range = 0 to 3.69]
Mean emotional eating	0.31 (0.39) [range = 0 to 3.02]
Within‐person centered emotional eating	0.00 (0.24) [range = −1.66 to 2.39]

As in prior studies examining hormone effects across the menstrual cycle (Klump et al. 2013, 2014, 2024), we excluded participants who only experienced flat or anovulatory cycles during the study (indicated by the absence of an estradiol peak and no luteal phase increase in progesterone on hormone plots independently coded by two raters; *n* = 43, 8.4% of original sample) because hormone effects may differ in such cycles (Asarian and Geary 2013) (see Supporting Information for a full description of menstrual phase coding methods). If participants had one ovulatory and one anovulatory cycle, data from their ovulatory cycle were included. Included and excluded participants did not significantly differ on mean emotional eating (*p* = 0.239, *d* = 0.19; Klump et al. 2024).

### 2.2 Procedure

As described in Klump et al. (2013), participants provided saliva samples for ovarian hormone measurements each morning (within 30 min of waking) and emotional eating ratings each evening (after 5 p.m.) daily for 45 consecutive days. This approach ensured hormone measures preceded behavioral ratings each day. Participants returned saliva samples and reconfirmed eligibility at three additional assessments at the beginning, midpoint, and end of data collection. Staff contacted participants once per week between assessments to answer questions and confirm protocol adherence. Dropout (3%) and missing data (≤ 6%) were minimal, and only a few (3%) participants became ineligible due to pregnancy or medication use (Klump et al. 2013).

### 2.3 Measures

#### 2.3.1 Emotional Eating

Emotional eating was assessed daily using the 13‐item Dutch Eating Behavior Questionnaire (DEBQ; van Strien et al. 1986) emotional eating subscale modified with permission to refer to that day. Past research has shown significant correlations between DEBQ emotional eating and other binge‐eating measures (e.g., van Strien 1996) and palatable food consumption in laboratory settings (van Strien 2000). Participants in the current study who endorsed objective binge‐eating episodes on the Structured Clinical Interview for DSM (First et al. 1996) had significantly higher average emotional eating scores than participants without binge eating (*d* = 0.84, *p* < 0.001; Klump et al. 2024). Importantly, within‐person fluctuations in ovarian hormones are robustly associated with within‐person phenotypic changes in DEBQ emotional eating across the menstrual cycle (Klump et al. 2008, 2013, 2014; Mikhail, Keel, et al. 2021). Internal consistency of the DEBQ emotional eating scale was excellent in this sample (average *α* = 0.90) and participants reported almost the full possible range of daily scores (range = 0–3.69; possible range = 0–4). Daily emotional eating scores were prorated if ≤ 10% of items were missing and marked as missing otherwise.

#### 2.3.2 Ovarian Hormones

Ovarian hormones were measured via daily saliva samples collected using previously reported methods (Klump et al. 2008, 2013). Saliva samples were assayed using highly sensitive enzyme immunoassay kits (Salimetrics LLC; see Supporting Information for additional details). To optimize resources, hormones were assayed from all samples collected during key periods of hormonal change (i.e., mid‐follicular through premenstrual phases) and every other day when hormone levels were expected to be low and stable (i.e., menstrual bleeding, early follicular phase). As previously reported (Klump et al. 2013, 2024), hormone levels followed the expected pattern of changes across the menstrual cycle (e.g., estradiol peaking during ovulation, progesterone peaking during the mid‐luteal phase; see Klump et al. 2013, 2014 for plotted graphs of within‐person hormone values across the cycle).

### 2.4 Statistical Analyses

#### 2.4.1 Data Transformations

As in previous research, daily hormone values were first transformed into five‐day rolling averages (e.g., Klump et al. 2013, 2014). Rolling averages are standard in hormone research with multiple daily measurements (Kassam et al. 1996; Waller et al. 1998) because they help account for potential delays in hormone effects on behavior (Eckel 2011) and reduce “noise” introduced through pulsatile hormone release (Bäckström et al. 1982; Gladis and Walsh 1987) and other forms of measurement error. Rolling averages have commonly been used in daily hormone studies of the menstrual cycle (Kassam et al. 1996; Santoro et al. 2003), disordered eating (Baker et al. 2019; Edler et al. 2007; Pang et al. 2023), and other psychiatric phenotypes (Eng et al. 2024; Peters et al. 2020; Owens et al. 2023; Ross et al. 2024). Hormone rolling averages were calculated if there were ≥ 3 days of data within the 5‐day window and counted as missing if there were < 3 days of data.

Daily emotional eating and hormone rolling averages were then within‐person centered (i.e., a participant's mean value was subtracted from each of their daily values). Following within‐person centering, hormone values were binned into person‐specific deciles (i.e., for each person, the lowest 10% of their values were assigned to decile 1, the next 10% to decile 2, etc.). This ensured sufficient observations at each level of the moderator to stably estimate genetic/environmental influences (Purcell 2002) while retaining within‐person ordering.

To investigate distinct within‐person effects, there must be meaningful day‐to‐day variability in both hormones and emotional eating from an individual's mean. We used mixed linear models to verify that a significant percentage of variance in daily emotional eating and rolling average ovarian hormone values could be attributed to within‐person fluctuations. As expected, we found significant within‐person variance, with approximately 31% of the variance in daily estradiol values, 42% of the variance in daily progesterone values, and 29% of the variance in daily emotional eating attributable to day‐to‐day fluctuations from an individual's mean (see Table 1 for ranges of within‐person centered values).

#### 2.4.2 Twin Models

Analyses used a variation of the van der Sluis et al. (2012) genotype x environment twin model that allows twins to differ on the moderator. This model examines differences in additive genetic (A; i.e., genetic influences that sum across genes), shared environmental (C; see Introduction), and nonshared environmental (E; i.e., environmental factors that differentiate co‐twins, including measurement error) influences on a variable across levels of a moderator. In the current study, this model tests whether twin similarity in day‐to‐day deviations in emotional eating from their own means depends on hormonal context, suggesting hormonally driven activation/suppression of genetic influences. Notably, this model rests on the assumption that hormones (moderators) are genetically independent of emotional eating (dependent variable). Otherwise, genetic mediation (i.e., gene–environment correlations) may be present. All genetic/environmental covariance between emotional eating and estradiol (Satorra‐Bentler χ^2^Δ (df) = 1.71 (3), *p* = 0.635) and progesterone (Satorra‐Bentler χ^2^Δ (df) = 3.98 (3), *p* = 0.264) could be constrained to zero, suggesting no significant shared genetic or environmental influences between hormones and emotional eating and eliminating the possibility of reverse causality (i.e., genetic/environmental influences on emotional eating influencing ovarian hormone levels).

As in Klump et al. (2024), to ensure adequate power and allow inclusion of quadratic as well as linear effects, initial analyses examined estradiol and progesterone separately in single moderator models. All models included nine parameters of interest: three path coefficients (a, c, e) that capture genetic/environmental influences when hormone levels are lowest and six moderation coefficients that capture linear (β_XH_, β_YH_, βz_H_) and quadratic (β_XH_^2^, β_YH_^2^, βz_H_^2^) changes in ACE as a function of hormone levels (see Figure S1). We first fit the full model with all paths and moderators freely estimated. We then fit submodels based on models that are commonly tested in twin research (e.g., constrain all A, C, or E moderators) and the full model results to identify a best‐fitting model. This approach allowed us to identify relevant submodels without conducting an excessive number of tests. Best‐fitting models were those that had a non‐significant difference in minus twice the log‐likelihood (−2lnL) between the full and nested model and minimized Akaike's Information Criterion (AIC), Bayesian Information Criterion (BIC), and sample‐size adjusted BIC (SABIC). If AIC, BIC, and SABIC identified different models as best‐fitting, the model that optimized two out of three indices and had a non‐significant change in −2lnL was selected. Following recommendations (Purcell 2002), tables and figures report unstandardized path coefficients and moderator estimates that reflect absolute differences in genetic/environmental influences across the moderators. Standardized estimates are also provided in the text to allow for comparisons with prior research (e.g., Klump et al. 2015, 2016, 2024).

Analyses were conducted in Mplus version 8.6 (Muthén and Muthén 1998) using robust full information maximum likelihood (FIML) estimation, which treats missing data as missing‐at‐random and is expected to produce less biased and more consistent estimates than other techniques (e.g., listwise deletion). Data were analyzed in “wide” format, in which each row contained observations for both twins in a family on a given study day. Analyses used the “cluster” function and Satorra‐Bentler scaled change in −2lnL (Satorra 2000), which accounts for clustering of daily observations within families. Because the robust FIML estimator also accounts for non‐normality, it was not necessary to log transform emotional eating. Analyses also controlled for whether a participant was menstruating on a given day (coded 0 = no, 1 = yes) and the day of participation (coded 1–45) to account for menstrual symptoms (e.g., pain) and reactivity (e.g., potential decreases in emotional eating over time due to monitoring) that could confound hormone effects.

## 3 Results

### 3.1 Estradiol Moderation

**FIGURE 1:**
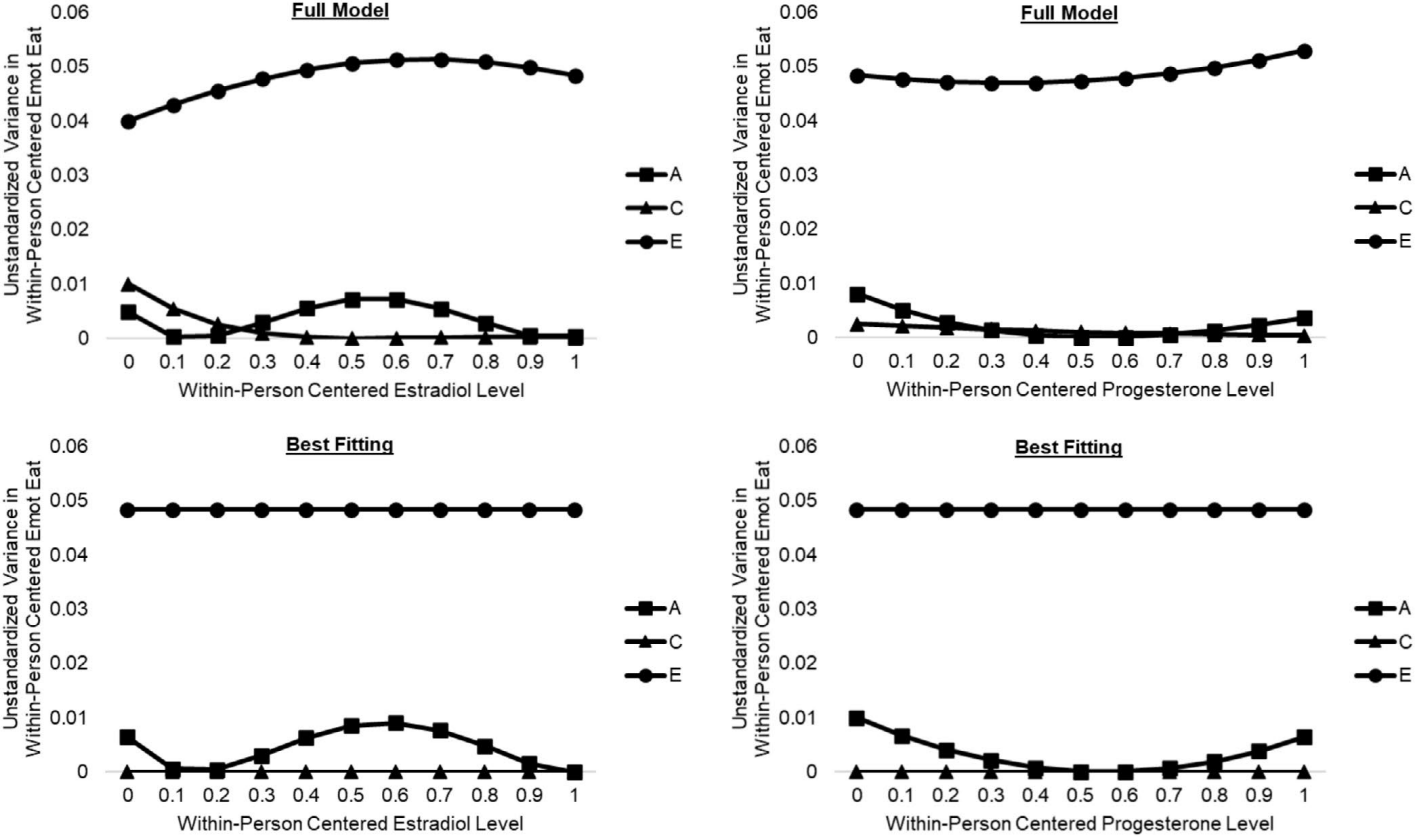
Genetic and environmental influences on within‐person emotional eating across within‐person estradiol and progesterone levels. A, additive genetic influences; C, shared environmental influences; E, nonshared environmental influences; Emot Eat, emotional eating. The *x*‐axis depicts within‐person centered hormone levels that were binned into person‐specific deciles for analyses.

Across all models, nonshared environmental influences had the greatest impact on within‐person fluctuations in emotional eating, which makes conceptual sense given that environmental factors that change from day to day (e.g., daily stressors; Fowler et al. 2023) are most likely to shape daily deviations in emotional eating from one's mean. Nevertheless, the full model also suggested genetic influences on within‐person changes in emotional eating that differed in a non‐linear manner across estradiol levels (see Figure 1). Genetic influences were highest when estradiol was moderate, with a second peak when estradiol was very low. Conversely, genetic influences decreased as estradiol levels increased beyond the midpoint, approaching zero when estradiol was at its within‐person peak. The full model also suggested more modest changes in environmental influences, with a potential decrease in shared environmental influences and an increase in nonshared environmental influences across estradiol levels.

Model‐fitting analyses confirmed these impressions. The best‐fitting model constrained the shared environmental main effect and moderation of shared and nonshared environmental influences to zero but retained moderation of genetic influences (see Table 2). This model fit best on two out of three fit indices and had a non‐significant change in −2lnL from the full model. Parameter estimates from the best‐fitting model (see Figure 1 and Table 3) showed genetic influences on within‐person fluctuations in emotional eating peaked when within‐person estradiol was moderate (accounting for ~16% of the variance), with a secondary peak when estradiol was very low (accounting for ~12% of the variance) (see Figure 1 and Table 3). Conversely, there were no detectable genetic influences on within‐person fluctuations in emotional eating when estradiol was at its within‐person peak (~0% of the variance).

**TABLE 2:** Model fit comparisons for within‐person hormone moderation of genetic/environmental influences on within‐person emotional eating.

Models	−2lnL	Scaling correction factor	−2lnLΔ (df)	*p*	AIC	BIC	SABIC
Single hormone models							
Estradiol models							
Full model	−1127.01	7.2001	—	—	−1093.01	−981.11	−1035.13
Nested submodels							
Constrain A quad and linear mods	−1121.41	6.9551	0.62 (2)	0.733	−1091.41	−992.67	−1040.34
Constrain C quad and linear mods	−1123.78	7.0774	0.40 (2)	0.819	−1093.78	−995.04	−1042.71
Constrain C quad mod	−1126.62	6.7273	0.03 (1)	0.862	−1094.62	−989.31	−1040.15
Constrain E quad and linear mods	−1119.16	6.5890	0.67 (2)	0.715	−1089.16	−990.42	−1038.09
Constrain E quad mod	−1123.22	6.3085	0.18 (1)	0.671	−1091.22	−985.90	−1036.75
Constrain all CE mods	−1116.21	7.5676	1.80 (4)	0.772	−1090.21	−1004.64	−1045.95
Constrain all CE mods, C main effect	**−1116.05**	**7.6601**	**1.80 (5)**	0.**876**	**−1092.05**	**−1013.06**	**−1051.20**
Constrain all mods	−1085.32	7.3176	5.97 (6)	0.427	−1063.32	−990.92	−1025.87
Progesterone models							
Full model	−1317.04	6.3302	—	—	−1283.04	−1170.73	−1224.75
Nested submodels							
Constrain A quad and linear mods	−1315.35	6.8059	0.61 (2)	0.737	−1285.35	−1186.25	−1233.91
Constrain C quad and linear mods	−1316.86	7.0157	0.15 (2)	0.928	−1286.86	−1187.76	−1235.43
Constrain E quad and linear mods	−1315.81	6.6891	0.34 (2)	0.844	−1285.81	−1186.71	−1234.38
Constrain all AC mods	−1311.25	7.2645	1.76 (4)	0.780	−1285.25	−1199.36	−1240.67
Constrain all CE mods	−1314.91	7.3666	0.72 (4)	0.949	−1288.91	−1203.02	−1244.33
Constrain all CE mods, A quad mod	−1314.78	7.6789	0.73 (5)	0.981	−1290.78	−1211.50	−1249.63
Constrain all CE mods, A quad mod, C main effect	**−1314.03**	**7.9735**	**0.91 (6)**	0.**989**	**−1292.03**	**−1219.36**	**−1254.31**
Constrain all mods	−1285.84	7.1609	6.49 (6)	0.371	−1263.84	−1191.17	−1226.12
Single hormone models, regressing out levels of the other hormone							
Estradiol models							
Full model	−1109.74	5.9067	—	—	−1075.74	−963.93	−1017.95
Nested submodels							
Constrain A quad and linear mods	−1109.45	6.5157	0.22 (2)	0.896	−1079.45	−980.79	−1028.46
Constrain C quad and linear mods	−1108.61	6.6446	3.05 (2)	0.218	−1078.61	−979.95	−1027.61
Constrain E quad and linear mods	−1106.71	6.2195	0.85 (2)	0.654	−1076.71	−978.05	−1025.72
Constrain E quad mod	−1106.81	5.9924	0.65 (1)	0.420	−1074.82	−969.58	−1020.42
Constrain all CE mods	−1105.57	6.9065	1.57 (4)	0.814	−1079.57	−994.07	−1035.38
Constrain all mods	−1104.64	7.1312	1.39 (6)	0.966	−1082.64	−1010.29	−1045.25
Constrain all mods, C main effect	**−1104.64**	**7.8445**	**1.63 (7)**	0.**977**	**−1084.64**	**−1018.87**	**−1050.65**
Progesterone models							
Full model	−1142.28	6.0464	—	—	−1108.28	−996.47	−1050.49
Nested submodels							
Constrain A quad and linear mods	−1142.00	6.4215	0.09 (2)	0.956	−1112.00	−1013.34	−1061.00
Constrain C quad and linear mods	−1142.19	6.4005	0.03 (2)	0.985	−1112.19	−1013.53	−1061.20
Constrain E quad and linear mods	−1136.74	6.2676	1.26 (2)	0.533	−1106.74	−1008.09	−1055.75
Constrain all AC mods	−1139.19	6.9706	1.01 (4)	0.908	−1113.20	−1027.69	−1069.00
Constrain all AC mods, E quad mod	−1116.05	7.1329	7.63 (5)	0.178	−1092.05	−1013.13	−1051.26
Constrain all AC mods, C main effect	**−1139.19**	**7.5515**	**1.27 (5)**	0.**938**	**−1115.20**	**−1036.27**	**−1074.40**
Constrain all CE mods	−1132.60	7.3716	5.57 (4)	0.234	−1106.60	−1021.10	−1062.41
Constrain all mods	−1115.80	6.9649	6.07 (6)	0.415	−1093.80	−1021.45	−1056.41
Estradiol‐to‐progesterone ratio models							
Full model	−1132.92	5.9718	—	—	−1098.92	−987.12	−1041.14
Nested submodels							
Constrain A quad and linear mods	−1130.89	6.6377	2.08 (2)	0.353	−1100.89	−1002.24	−1049.90
Constrain C quad and linear mods	−1131.31	6.6447	1.74 (2)	0.419	−1101.31	−1002.66	−1050.32
Constrain all C mods, C main effect	−1131.31	7.1193	2.62 (3)	0.454	−1103.31	−1011.23	−1055.72
Constrain all C mods, C main effect, A quad mod	−1126.71	7.2846	3.65 (4)	0.455	−1100.71	−1015.20	−1056.51
Constrain all C mods, C main effect, E quad mod	−1127.80	7.1010	2.22 (4)	0.695	−1101.80	−1016.30	−1057.61
Constrain E quad and linear mods	−1125.89	6.1624	1.55 (2)	0.461	−1095.89	−997.23	−1044.90
Constrain all CE mods	−1124.88	6.8700	2.63 (4)	0.622	−1098.88	−1013.38	−1054.69
Constrain all CE mods, C main effect	**−1124.88**	**7.4426**	**3.29 (5)**	0.**655**	**−1100.88**	**−1021.96**	**−1060.09**
Constrain all mods	−1105.59	6.9615	6.57 (6)	0.362	−1083.59	−1011.25	−1046.20

*Note*: Dashes indicate parameters are not applicable. The best‐fitting model is bolded.

Abbreviations: −2lnL, minus twice the log‐likelihood; −2lnLΔ, change in −2lnL; A, additive genetic influences; AIC, Akaike Information Criterion; BIC, Bayesian Information Criterion; C, shared environmental influences; df, degrees of freedom; E, nonshared environmental influences; mod, moderator; SABIC, sample size adjusted Bayesian Information Criterion.

**TABLE 3:** Parameter estimates from the full and best‐fitting genotype × hormone models.

Models	a	Linear a mod	Quad a mod	c	Linear c mod	Quad c mod	e	Linear e mod	Quad e mod
Single hormone models									
Estrogen models									
Full model	0.07 (−0.15, 0.29)	−0.57 (−1.31, 0.17)	0.52 (−0.10, 1.15)	0.10 (−0.07, 0.26)	−0.28 (−1.71, 1.16)	0.17 (−1.76, 2.09)	**0.20 (0.17, 0.23)**	0.08 (−0.07, 0.23)	−0.06 (−0.20, 0.09)
Best fitting	**−0.08 (−0.14, −0.03)**	**0.61 (0.30, 0.92)**	**−0.53 (−0.88, −0.19)**	—	—	—	**0.22 (0.20, 0.24)**	—	—
Progesterone models									
Full model	−0.09 (−0.23, 0.06)	0.19 (−0.17, 0.55)	−0.04 (−0.38, 0.29)	0.05 (−0.14, 0.24)	−0.04 (−0.45, 0.36)	0.01 (−0.35, 0.37)	0.**22 (0.19, 0.25)**	−0.02 (−0.14, 0.09)	0.03 (−0.07, 0.13)
Best fitting	0.**10**	**−0.18**	—	—	—	—	0.**22**	—	—
	**(0.06, 0.14)**	**(−0.28, −0.09)**					**(0.20, 0.24)**		
Single hormone models, regressing out levels of the other hormone									
Estrogen models									
Full model	−0.07 (−0.18, 0.03)	0.10 (−0.24, 0.45)	−0.06 (−0.27, 0.15)	−0.05 (−0.22, 0.12)	0.14 (−0.36, 0.64)	−0.04 (−0.50, 0.42)	**0**.**21 (0.19, 0.24)**	0.06 (−0.05, 0.16)	−0.05 (−0.16, 0.05)
Best fitting	**0**.**05 (0.01, 0.09)**	—	—	—	—	—	**0**.**22 (0.20, 0.25)**	—	—
Progesterone models									
Full model	0.09 (−0.11, 0.28)	−0.18 (−1.25, 0.89)	0.13 (−0.67, 0.92)	0.03 (−0.40, 0.46)	−0.15 (−1.65, 1.35)	0.12 (−1.01, 1.25)	**0**.**23 (0.19, 0.26)**	−0.04 (−0.21, 0.13)	0.05 (−0.08, 0.19)
Best fitting	**0**.**05 (0.01, 0.09)**	—	—	—	—	—	**0**.**24 (0.21, 0.27)**	**−0.09 (−0.17, −0.01)**	**0**.**09 (0.01, 0.17)**
Estradiol‐to‐progesterone ratio models									
Full model	**−0.05 (−0.09, −0.01)**	−0.14 (−0.39, 0.12)	0.20 (−0.07, 0.47)	0.02 (−0.03, 0.08)	−0.19 (−0.56, 0.19)	0.23 (−0.15, 0.62)	**0**.**21 (0.18, 0.24)**	0.08 (−0.07, 0.22)	−0.07 (−0.22, 0.08)
Best fitting	−0.02 (−0.08, 0.03)	−0.28 (−0.65, 0.09)	0.33 (−0.10, 0.75)	—	—	—	**0**.**22 (0.20, 0.25)**	—	—

*Note*: Significant model parameters are bolded, with the 95% CI in parentheses.

Abbreviations: a, additive genetic influences; c, shared environmental influences; e, nonshared environmental influences; mod, moderator; quad, quadratic.

### 3.2 Progesterone Moderation

Moderation of genetic/environmental influences on emotional eating by within‐person changes in progesterone were somewhat more subtle (see Figure 1) but suggested potentially stronger genetic influences at high and low levels of progesterone relative to moderate levels. In model fitting analyses, the best‐fitting model constrained all shared environmental influences, moderation of shared and nonshared environmental influences, and quadratic moderation of genetic influences to zero, but retained linear moderation of genetic influences (see Table 2). This model was preferred on all three fit indices and showed a non‐significant change in −2lnL. Because genetic influences started above zero at low progesterone levels, the linear genetic moderation effect created a pattern in which genetic influences were high when progesterone was low (accounting for ~17% of variance in emotional eating), decreased to zero when progesterone was moderate, then increased again when progesterone was high (accounting for ~12% of variance) (see Figure 1 and Table 3).

### 3.3 Follow‐Up Models of Estrogen and Progesterone Interplay

Although individual hormone analyses provide initial indication of etiologic moderation, the natural covariation between estradiol and progesterone (*r* = 0.36, *p* < 0.001 in this sample) highlights the need for models that also capture their joint influence. Follow‐up models were conducted to further elucidate these effects.

**FIGURE 2:**
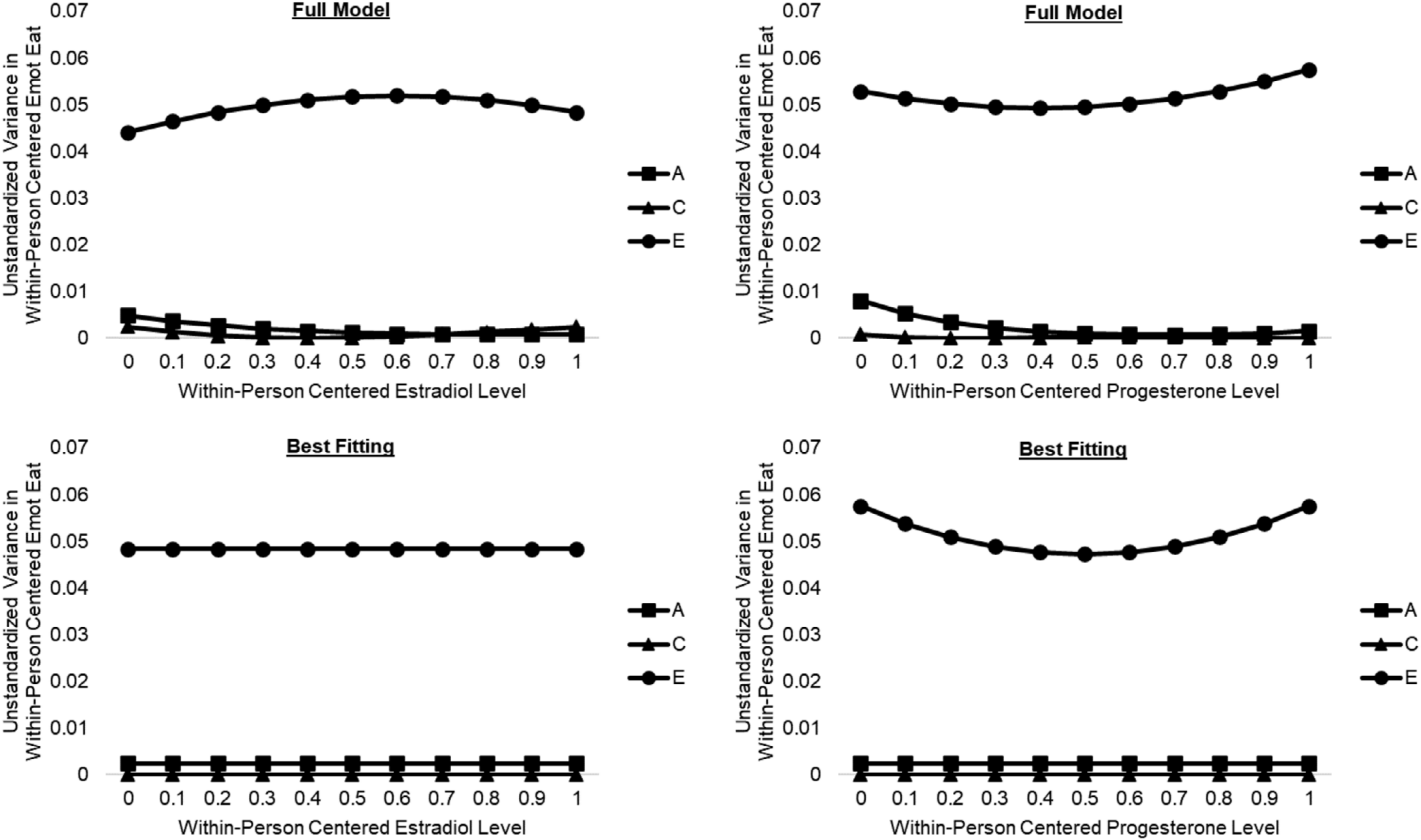
Genetic and environmental influences on within‐person emotional eating across within‐person estradiol and progesterone levels, with levels of the other hormone regressed out. A, additive genetic influences; C, shared environmental influences; E, nonshared environmental influences; Emot Eat, emotional eating. The *x*‐axis depicts within‐person centered hormone levels that were binned into person‐specific deciles for analyses.

We first conducted analyses that regressed levels of one hormone out from the other (e.g., regressed estradiol levels out of progesterone in progesterone analyses) to attempt to identify hormone‐specific effects. Levels of one hormone were regressed out of the other in twin 1 and twin 2 prior to creating person‐specific deciles to ensure enough observations remained at each level of the moderator. Moderation effects were much smaller after regressing out levels of the other hormone (see Figure 2 and Table 3). All moderation of genetic influences could be constrained to zero for both hormones without a decrement in model fit, and the no moderation model provided the best fit for estradiol (see Table 2). The best‐fitting model for progesterone retained some moderation of nonshared environmental influences; however, the change in the proportion of variance due to nonshared environmental factors across progesterone levels was small (see Table 3 and Figure 2). These findings suggest that the within‐person effects of estradiol and progesterone may not be fully separable.

To better understand how estradiol and progesterone may work together to shift genetic/environmental influences, we conducted additional analyses examining the combination of hormones present at a given time. We considered fitting double moderator, estradiol × progesterone models; however, simulation studies suggest substantially larger samples are needed for these models (Burt et al. 2020; Purcell 2002). Moreover, we observed non‐linear changes in etiologic effects in single hormone models, and double moderator models do not easily allow for non‐linear changes. Instead, we fit models that examined the ratio of estradiol to progesterone on a given day. Use of a single ratio value as the moderator maximizes power and allows for non‐linear effects.

**FIGURE 3:**
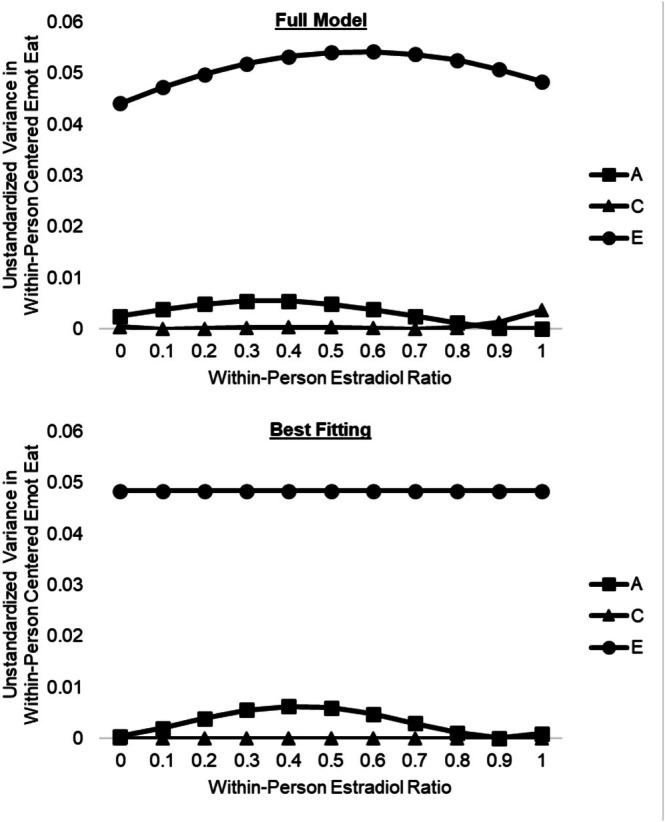
Genetic and environmental influences on within‐person emotional eating across within‐person estradiol‐to‐progesterone ratio. A, additive genetic influences; C, shared environmental influences; E, nonshared environmental influences; Emot Eat, emotional eating. The *x*‐axis depicts within‐person centered estradiol‐to‐progesterone ratios that were binned into person‐specific deciles for analyses.

The full ratio model (see Figure 3) suggested that genetic and nonshared influences were greatest when the estradiol‐to‐progesterone ratio was moderate, whereas shared environmental influences increased at higher ratios only. The best‐fitting model retained moderation of genetic influences but constrained all shared environmental influences and moderation of shared and nonshared environmental influences to zero (See Figure 3 and Table 2). This model was preferred on two out of three fit indices and had a non‐significant change in −2lnL. Like the full model, it suggested that genetic influences were greater when the estradiol‐to‐progesterone ratio was moderate (accounting for ~11% of the variance) versus low or high (< 2% of the variance). However, these results should be interpreted with some caution given that neither linear nor quadratic moderation of genetic influences was statistically significant in the best‐fitting model despite the fact that model fit indices suggested these parameters should be retained.

## 4 Discussion

To our knowledge, this is the first study to investigate how within‐person fluctuations in ovarian hormones may impact genetic and environmental influences on day‐to‐day changes in any behavior from an individual's baseline. Results have important implications for our understanding of factors that may contribute to shifts in dysregulated eating across days and the menstrual cycle. Specifically, the extent to which within‐person changes in emotional eating were driven by genetic influences differed across within‐person estradiol and progesterone levels, suggesting that dynamic hormone regulation of gene expression may underlie phasic changes in dysregulated eating. Crucially, the hormonal milieus associated with increased genetic influences on changes in emotional eating (i.e., moderate estradiol/high progesterone) map onto the menstrual cycle phase previously identified as riskiest for phenotypic increases in dysregulated eating (i.e., mid‐luteal phase; Klump et al. 2013), supporting a link between activation of genetic influences and phenotypic symptom increases. Results highlight the critical importance of modeling phasic, within‐person hormone effects on etiology and behavior, as well as the potentially key role of hormones in shaping expression of genetic risk for dysregulated eating across the menstrual cycle.

Analyses of the estradiol‐to‐progesterone ratio suggested that rather than each hormone acting in isolation, it may be the combination of ovarian hormone levels at critical points in the menstrual cycle that drives expression of genes related to dysregulated eating. This interpretation is supported by analyses that regressed levels of one hormone out from the other, which showed that moderation by each hormone was substantially reduced when examined completely independently of the other's influence (see Tables 2 and 3 and Figure 2). Because progesterone often exerts its effects by antagonizing those of estradiol (Eckel 2011), hormonal milieus characterized by moderate within‐person estradiol and high within‐person progesterone (i.e., mid‐luteal) may “uncover” genes whose influence on dysregulated eating is suppressed by high estradiol when progesterone is no more than moderate. Notably, this pattern is consistent with phenotypic studies showing a significant interaction between within‐person estradiol and progesterone across the menstrual cycle in predicting within‐person changes in emotional eating (Klump et al. 2013). Although we did not have adequate power to directly model interactions between estradiol and progesterone in the current study, this is an important area for future research, particularly as modeling techniques continue to evolve.

Distinct within‐person hormone effects may facilitate increased precision in the timing of reproductively relevant behavior, including eating behavior (Schneider et al. 2013). Shifts in motivation away from food and toward sexual behavior during the period of maximum fertility (i.e., ovulation) have been proposed to promote reproductive success (Asarian and Geary 2013; Eckel 2011; Schneider et al. 2013). This Motivational Priority Shifts Hypothesis (Roney and Simmons 2017; Schleifenbaum et al. 2024) provides an evolutionary rationale for phasic regulation of eating behavior by ovarian hormones through changes in gene expression. Individuals may be particularly likely to experience dysregulated eating during “risky” hormonal milieus if they have a higher loading for hormonally regulated genes related to appetitive drive, leading to loss of control over eating when these genes are expressed. Notably, the pattern of etiologic moderation was largely unchanged when negative affect (measured daily using the Positive and Negative Affect Schedule; Watson et al. 1988) was regressed out of emotional eating in supplemental analyses (see Table S1 and Figure S2), supporting the idea that effects are more likely to be related to appetitive and/or reward processes (e.g., regulation of hunger/satiety, responsiveness to food cues, liking of food consumed) rather than ovarian hormone influences on more distal factors such as mood.

Interestingly, the patterns of etiologic moderation in this study were somewhat distinct from those of mean and absolute daily hormone levels (Klump et al. 2016, 2024), particularly for estradiol, suggesting unique within‐person hormone effects. While higher mean and raw daily estradiol levels increase shared environmental influences on dysregulated eating (Klump et al. 2016, 2024), results from this study show that within‐person estradiol fluctuations moderate genetic influences. Moreover, in past work, absolute estrogen and progesterone levels had fully independent effects on genetic/environmental influences on emotional eating (Klump et al. 2024). That was not the case in the current study, suggesting a more complicated and dynamic interplay between estrogen and progesterone at the within‐person level. They also raise the interesting possibility that estrogen may have more than one mode of action on emotional eating in women—with potential non‐genomic effects (e.g., via membrane estrogen receptors; see Klump et al. 2024) impacting overall levels of emotional eating and largely genetic influences (in concert with progesterone) on changes in emotional eating across days and menstrual cycle phase. Additional research is needed to investigate these possibilities and more precisely disentangle these types of genomic and non‐genomic effects. Animal models that can directly manipulate within‐individual changes in hormone levels and measure gene expression and neurotransmitter activity within the brain would be ideal for exploring the precise mechanisms of hormone effects in future work.

Before closing, some limitations should be noted. Although representative of Michigan in race and ethnicity, our sample nevertheless had somewhat limited representation of individuals of color and people from socioeconomically disadvantaged backgrounds. It is critical to examine whether effects are similar in more diverse samples, particularly given evidence that socioeconomic disadvantage may moderate genetic influences on disordered eating (Burnette et al. 2024; Mikhail, Carroll, et al. 2021). Participants were also relatively young, and it would be interesting to examine whether etiologic hormone effects are similar in older participants (e.g., women approaching menopause). Our use of a non‐clinical, regularly menstruating sample allowed us to maximize power and variability in dysregulated eating across participants, which is critical for twin models that parse variability across a population. However, results may not fully generalize to clinical samples with the most severe dysregulated eating or to individuals with ovarian hormone dysregulation, which is more prevalent among women with clinically significant binge eating (Cooney et al. 2024).

There are other methods for analyzing repeated‐measures twin data, including longitudinal biometric mixed‐effects models (McArdle 2006) and longitudinal Cholesky decomposition models (e.g., Nas et al. 2025), that could offer additional insights into the time‐varying influence of ovarian hormones on dysregulated eating. Although the expected changes in estradiol and progesterone across the menstrual cycle were present in over 90% of twins as verified by two independent raters (and twins without these changes were excluded from present analyses), all hormone research provides only an estimation of hormone levels and is subject to measurement error (van Anders et al. 2014), which could reduce our sensitivity or precision in detecting hormone effects. Future research should aim to use multiple hormone assay methods (e.g., enzyme immunoassays, LC–MS/MS) to further validate hormone/behavior results, especially given the unique limitations of each method (e.g., increased data loss with LC–MC/MS; Brouillard et al. 2025). Additionally, we had to rely on estradiol: progesterone ratios for exploration of hormone interplay due to power constraints. While ratios provide some insight into these interactive processes, results involving ratios should be interpreted with caution because they do not account for absolute hormone levels (in other words, low estradiol/low progesterone and high estradiol/high progesterone are treated as equivalent; Sollberger and Ehlert 2016). This is particularly important given the pattern of complex, non‐linear, and possibly dependent hormone effects observed in this study.

## Author Contributions

**Megan E. Mikhail:** conceptualization, investigation, writing – original draft, methodology, visualization, formal analysis. **Kristen M. Culbert:** writing – review and editing, conceptualization. **Pamela K. Keel:** conceptualization, methodology, writing – review and editing, funding acquisition. **S. Alexandra Burt:** conceptualization, methodology, writing – review and editing, funding acquisition. **Cheryl L. Sisk:** conceptualization, methodology, writing – review and editing, funding acquisition. **Alexander Johnson:** writing – review and editing, conceptualization. **Steven Boker:** conceptualization, methodology, writing – review and editing, funding acquisition. **Micheal C. Neale:** conceptualization, methodology, writing – review and editing, funding acquisition. **Kelly L. Klump:** conceptualization, methodology, funding acquisition, writing – review and editing, supervision, project administration.

## Ethics Statement

Study procedures were approved by the Michigan State University Institutional Review Board.

## Conflicts of Interest

The authors declare no conflicts of interest.

## Supporting information


**Data S1:** eat24545‐sup‐0001‐Supinfo.docx.

## Data Availability

The data that support the findings of this study are available from the corresponding author upon reasonable request.
