# Supplementary material for: Within‐Person Changes in Daily Ovarian Hormone Levels Influence Genetic Effects on Emotional Eating in Women
Source: Int J Eat Disord. 2025 Sep 17;58(12):2352–65. doi: 10.1002/eat.24545 (PMC12703227; doi:10.1002/eat.24545)
Supplement: Supplementary file 1 — Data S1: eat24545‐sup‐0001‐Supinfo.docx. [file EAT-58-2352-s001.docx]

**Supplemental Material**

**Additional Hormone Measurement Details**

Saliva samples were assayed using specialized, highly sensitive enzyme immunoassay kits (Salimetrics, LLC) that show excellent reliability (intra- and inter-assay coefficients of variation: estradiol = 7.1% and 7.5%; progesterone = 6.2% and 7.6%), specificity (determined by interpolating the mean optical density minus 2 SD of 10-20 replicates at the 0 pg/ml level; estradiol = .10 pg/ml; progesterone = 5 pg/ml), and method accuracy (measured via spike recovery and linearity; estradiol = 104.2% and 99.4%; progesterone = 99.6% and 91.8%). Cross-reactivity with similar hormones is also low (e.g., <1.3% for estradiol with estriol, estrone, ethynylestradiol, etc.; <0.2% for progesterone with prednisolone, prednisone, cortisone, etc.).

Anovulatory cycles were identified through visual inspection of hormone plots, which was completed by two independent raters for every participant. Specifically, every twin’s levels of estradiol and progesterone were individually plotted across the 45-day study period and independently coded by two research assistants into cycle phases (follicular, ovulatory, mid-luteal, premenstrual, menstrual bleeding) based on recorded days of menstrual bleeding and observed hormone changes (see Edler et al., 2007 for additional details regarding coding methods). All raters underwent extensive training and were required to achieve an inter-rater reliability of ≥0.80 with senior raters for cycle phase coding (Klump et al., 2015). The small percentage (8.4%) of menstrual cycles that did not show the expected pattern of ovarian hormone changes, i.e., an estradiol peak in the late follicular phase followed by a substantial increase in progesterone in the post-ovulatory/luteal phase, were labeled anovulatory and excluded from current analyses.

Saliva samples represent a more feasible method of hormone collection across an extended timeframe and show higher compliance rates and stronger hormone-behavior associations than more invasive techniques (e.g., bloodspots) (Edler et al., 2007). Use of a repeated sampling approach (e.g., daily saliva samples across 45 days of data collection) that resulted in a large number of samples per participant also reduces concerns regarding error that may be present with only one or a limited number of samples.

**Table S1.** Full model parameter estimates controlling for negative affect.

| Models | **a** | **Linear**  **a mod** | **Quad**  **a mod** | **C** | **Linear**  **c mod** | **Quad**  **c mod** | **e** | **Linear**  **e mod** | **Quad**  **e mod** |  |
| --- | --- | --- | --- | --- | --- | --- | --- | --- | --- | --- |
| **Single Hormone Models** | | | | | | | | | |  |
| **Estrogen Model** |  |  |  |  |  |  |  |  |  |  |
|  | .04  (-.05, .13) | **-.48**  **(-.82, -.14)** | **.43**  **(.10, .76)** | **.11**  **(.06, .17)** | **-.44**  **(-.77, -.10)** | **.39**  **(.02, .76)** | **.20**  **(.17, .22)** | .08  (-.07, .24) | -.07  (-.22, .09) |  |
|  |  |  |  |  |  |  |  |  |  |  |
| **Progesterone Model** | | | | | | | | | |  |
|  | -.07  (-.42, .27) | .17  (-.28, .61) | -.03  (-.38, .33) | .07  (-.17, .32) | -.09  (-.65, .47) | .04  (-.31, .39) | **.22**  **(.17, .26)** | -.004  (-.16, .16) | .01  (-.11, .13) |  |
| **Single Hormone Models, Regressing Out Levels of the Other Hormone** | | | | | | | | | |  |
| **Estrogen Model** | |  |  |  |  |  |  |  |  |  |
|  | | **-.08**  **(-.13, -.02)** | .10  (-.10, .31) | -.08  (-.29, .12) | -.04  (-.16, .08) | .11  (-.34, .56) | -.03  (-.47, .42) | **.21**  **(.19, .24)** | .05  (-.06, .15) | -.04  (-.15, .06) |
|  | |  |  |  |  |  |  |  |  |  |
| **Progesterone Model** | | | | | | | | | | |
|  | | -.09  (-.32, .14) | .12  (-.91, 1.16) | -.08  (-.87, .70) | -.05  (-.39, .28) | .21  (-.80, 1.23) | -.16  (-.91, .58) | **.22**  **(.19, .25)** | -.03  (-.18, .12) | .04  (-.08, .17) |
|  | |  |  |  |  |  |  |  |  |  |
| **Estradiol-to-Progesterone Ratio Model** | | | | | | | | | |  |
|  | **-.06**  **(-.09, -.02)** | -.12  (-.36, .11) | .18  (-.07, .42) | .02  (-.03, .07) | -.18  (-.53, .18) | .23  (-.15, .61) | **.21**  **(.18, .23)** | .06  (-.08, .20) | -.06  (-.21, .09) |  |

Note. All models controlled for negative affect assessed with the Positive and Negative Affect Schedule. a = additive genetic influences; c = shared environmental influences; e = nonshared environmental influences; mod = moderator; quad = quadratic. Significant model parameters are bolded, with the 95% CI in parentheses.

**
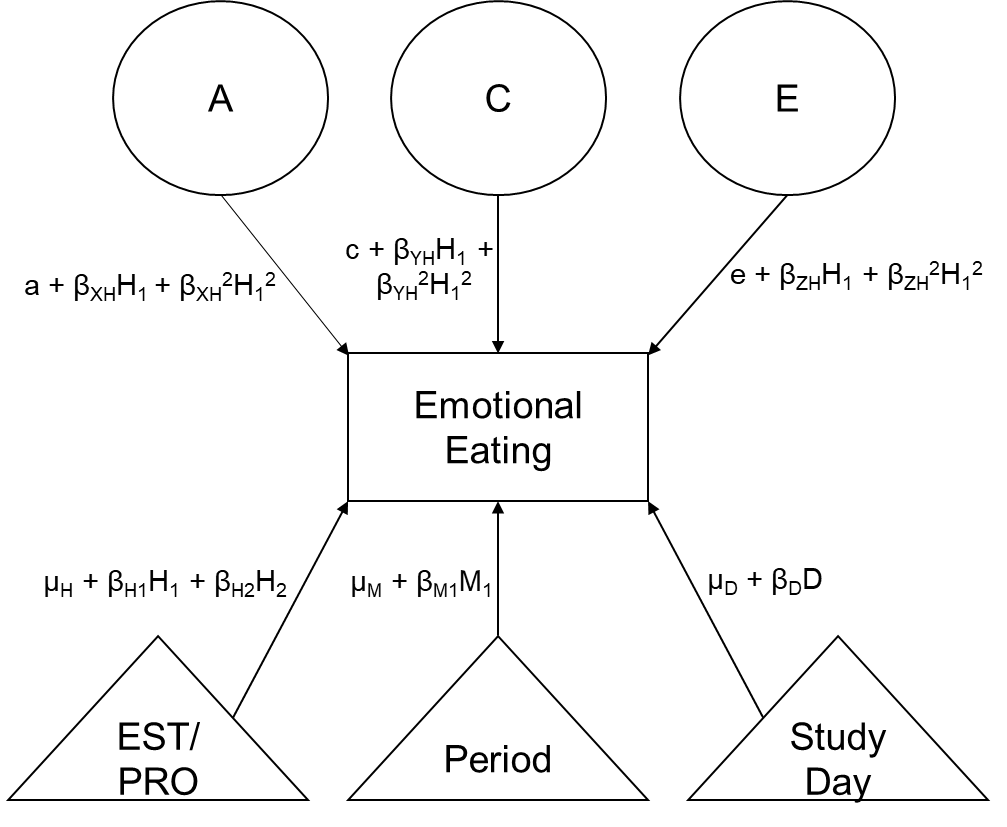
**

**Figure S1.** Path diagram for the full within-person genotype x hormone moderation model. Emotional eating = within-person centered emotional eating; EST/PRO = within-person centered estradiol, progesterone, or estradiol-to-progesterone ratio; period = whether the participant experienced menstrual bleeding that day; study day = day of participation in the study; A = additive genetic influences; C = shared environmental influences; E = nonshared environmental influences; H_1_ and H_2_ = hormone levels for twin 1 and twin 2; M_1_ = whether twin 1 was menstruating on a given day (coded 0 or 1); D = day of participation; μ_H_, μ_M_, μ_D_, a, c, e = intercepts; β_H1_ = regression coefficient representing the phenotypic association between twin 1’s hormone levels and their own emotional eating; β_H2_ = regression coefficient representing the phenotypic association between twin 2’s hormone levels and twin 1’s emotional eating; β_D_ = regression coefficient representing the phenotypic association between study day and twin 1’s emotional eating; β_M1_ = regression coefficient representing the phenotypic association between twin 1’s menstrual status and their own emotional eating; β_XH_, β_YH_, β_ZH_ = coefficients for linear moderation of genetic and environmental influences by hormone levels; β_XH_^2^, β_YH_^2^, β_ZH_^2^ = coefficients for quadratic moderation of genetic and environmental influences by hormone levels.


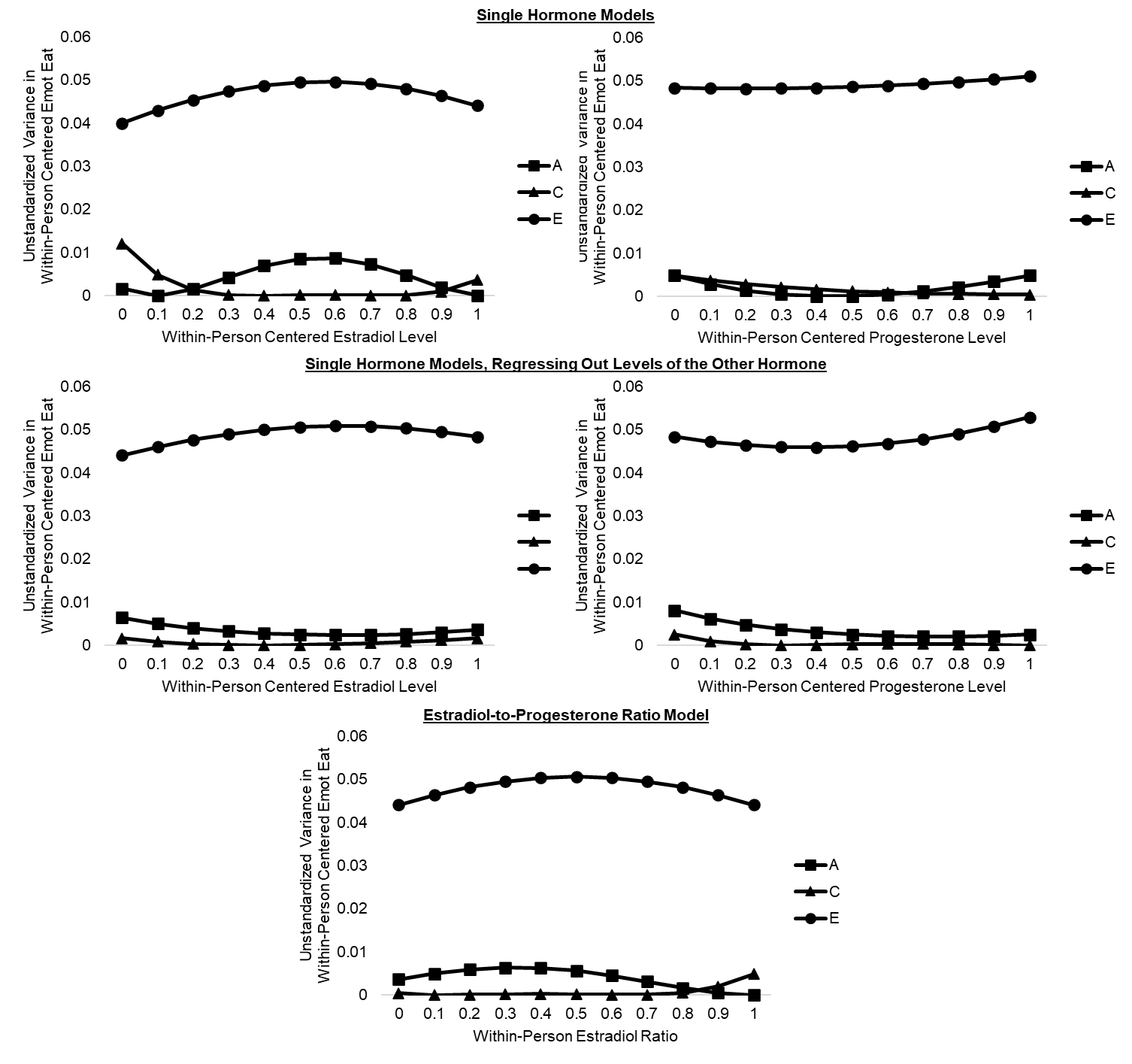


**Figure S2.** Full models for genetic and environmental influences on within-person emotional eating across within-person estradiol and progesterone levels, controlling for negative affect. All models controlled for negative affect assessed with the Positive and Negative Affect Schedule. A = additive genetic influences; C = shared environmental influences; E = nonshared environmental influences; Emot Eat = emotional eating. The x-axis depicts within-person centered hormone levels that were binned into person-specific deciles for analyses.
